# Filamentary Convolution for SLI: A Brain-Inspired Approach with High Efficiency

**DOI:** 10.3390/s25103085

**Published:** 2025-05-13

**Authors:** Boyuan Zhang, Xibang Yang, Tong Xie, Shuyuan Zhu, Bing Zeng

**Affiliations:** 1 School of Information and Communication Engineering, University of Electronic Science and Technology of China, Chengdu 611731, China; 202011012231@std.uestc.edu.cn (B.Z.); 202221011822@std.uestc.edu.cn (X.Y.); eezsy@uestc.edu.cn (S.Z.); 2 Nanyang Technological University, 50 Nanyang Avenue Avenue, Singapore 639798, Singapore; xiet0004@e.ntu.edu.sg

**Keywords:** spoken language identification, deep learning network (DLN), filamentary convolution, frequency-level feature extraction

## Abstract

Spoken language identification (SLI) relies on detecting key frequency characteristics like pitch, tone, and rhythm. While the short-time Fourier transform (STFT) generates time–frequency acoustic features (TFAF) for deep learning networks (DLNs), rectangular convolution kernels cause frequency mixing and aliasing, degrading feature extraction. We propose filamentary convolution to replace rectangular kernels, reducing the parameters while preserving inter-frame features by focusing solely on frequency patterns. Visualization confirms its enhanced sensitivity to critical frequency variations (e.g., intonation, rhythm) for language recognition. Evaluated via self-built datasets and cross-validated with public corpora, filamentary convolution improves the low-level feature extraction efficiency and synergizes with temporal models (LSTM/TDNN) to boost recognition. This method addresses aliasing limitations while maintaining computational efficiency in SLI systems.

## 1. Introduction

Humans typically rely on discerning individual words or short voice clips within a spoken language signal, especially when encountering an unfamiliar language. This process is facilitated by top-down auditory sensing, as suggested by studies in auditory neuroscience [1], where the speech signal undergoes hierarchical transduction and decoupling to extract key information in the human brain.

Research in auditory neuroscience indicates that neurons in the brain cortex initially convert sound into frequency information, aiding in the extraction of useful information through frequency-based filtering. Moreover, during language acquisition, the auditory pathway is primarily shaped by primary frequency stimuli, such as tone, pitch, and loudness [2].

To capture the short-time spectral frequency information inherent in human auditory perception, the short-time Fourier transform (STFT) is commonly used to generate time–frequency joint features (TFAFs) for audio signals. These TFAFs are extensively utilized in spoken language identification (SLI) systems, including deep learning networks (DLNs). The windowing mechanism in the STFT is designed to encapsulate characteristics into corresponding windows, adhering to the “short-term unit” principle, which facilitates the frame-by-frame extraction of TFAFs. Each window’s frequency spectrum constitutes a frame, offering comprehensive time–frequency joint characteristics for identification. This enables subsequent SLI systems to perform accurate classification more easily.

**Figure 1:**
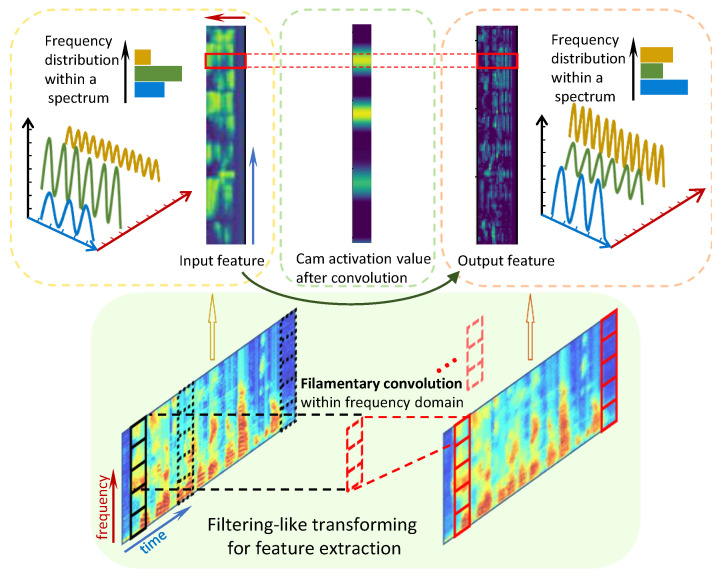
Feature extraction for filamentary convolution.

However, many existing DLN-based SLI systems employ rectangular-shaped convolution kernels that span multiple adjacent frames. These kernels blend cross-frame information and consolidate multiple frames into a single integrated unit, which contradicts human hearing and understanding mechanisms, particularly the filtering effect [3,4]. To mitigate this issue, we propose filamentary convolution, which utilizes filamentary-shaped kernels to conduct convolution within a single frame, aligning with the human filtering effect. This “filter-like” effect facilitates feature extraction and encoding within the frequency domain. As depicted in Figure 1, filamentary convolution can execute parallel feature extraction while preserving frequency information across different time scales.

In this study, we elucidate the fundamental distinctions between filamentary convolution and rectangular-shaped convolution. Through a comparative analysis of the two convolution methods, we demonstrate that rectangular-shaped convolution exhibits redundancy. Additionally, we contrast filamentary convolution with one-dimensional (1-D) time convolution. By comparing the two uncoupled convolution methods, we demonstrate that incorporating cross-frame operations into TFAF low-level feature extraction results in degradation in the language recognition performance. This finding substantiates the fact that the premature integration of temporal information poses challenges to language identification. Conversely, filamentary convolution yields an enhancement in the language feature extraction performance. Furthermore, we conduct experiments on self-constructed datasets, with particular experiments aimed at visual validation. The experimental results demonstrate that filamentary convolution outperforms the other methods in efficiency by capturing frame-level characteristics and maintaining frame-level relationships, which are conducive to the subsequent extraction of crucial temporal information. These advantages render filamentary convolution more efficient for feature extraction in language identification tasks, and it can be widely employed as a highly effective raw feature extractor.

## 2. Related Work

This work focuses on demonstrating the higher efficiency of filamentary convolution compared to traditional convolutional methods in SLI. Although language identification is our primary validation scenario as a classification task for speech signals, we perform further validation on other classification models, such as models in automatic speaker recognition (ASR), etc.

### 2.1. Convolution for Spoken Language Identification

Convolutional operations are adept in efficient computation over multidimensional features and are tailored to adapt to various scenarios. The most basic form of convolution is rectangular-shaped convolution, which serves as a foundational element for further adaptive designs. Some studies delve into the design of convolution kernels to suit specific feature extraction requirements in different contexts. For instance, three-dimensional convolution is proposed for spatiotemporal feature learning to accommodate the three-dimensional representation of video signals [5]; dilated convolution is designed to preserve the spatial resolution and increase the receptive field in image classification [6]; and deformable convolution incorporates additional learnable offsets to enhance the transformation modeling capabilities [7]. Other convolutional approaches aim at improving the efficiency: group convolution, initially proposed in AlexNet [8], enables more efficient GPU implementation; depthwise separable convolutions, introduced in [9], consist of a depthwise convolution followed by a pointwise convolution and have been found to be more efficient than the basic Inception architecture [10].

Rectangular-shaped convolution, as discussed in [11,12,13], has demonstrated strong compatibility with TFAFs in SLI tasks. Further exploration in the LID-net system [12] restricted convolution covering the entire frequency domain to one-dimensional convolution for information extraction within the frequency domain. A recent seminal work [14] in ASR delved into the impact of stride-induced temporal and frequency resolution changes on the final efficiency within the convolutional framework. The study concluded that preserving the temporal resolution often leads to higher efficiency. These insights potentially prove filamentary-shaped convolution to be a more efficient feature extraction strategy than traditional ones, while causal disentanglement methods (e.g., [15]) address cross-silo data challenges in feature representation.

### 2.2. Deep Learning-Based Frameworks for Spoken Language Identification

Due to the powerful computational capabilities of deep learning, it has been widely applied in the fields of SLI and ASV. Initially, DNN-based methods [16,17] were applied to learn discriminative language information from speech signals for effective identification. Subsequently, convolutional neural networks (CNNs), particularly Time-Delay Neural Networks (TDNNs) [11], were employed to extract temporal features for language classification. A hybrid DNN-CNN method was proposed in [12], similar to the CNN-LSTM approach in [18]. Moreover, from recurrent neural networks (RNNs) to long short-term memory recurrent neural networks (LSTMs), memory mechanism-based networks have exhibited superiority in the SLI task; for example, LSTM-based LID systems [19,20,21] have achieved high accuracy in language identification. Recently, attention mechanisms have been integrated into LSTM-based [22] and gated recurrent unit (GRU)-based [23] SLI systems to enhance temporal frame correlation and select informative features. Subsequent studies have focused on frequency-domain feature extraction. Time–frequency attention mechanisms [13] have been applied to spectral sequences to discern language features in both the time and frequency domains. Furthermore, SLI systems with a larger parameter count, such as those employing attention and parallel structures, have been developed. In PHO-LID [24], the single identification flow is expanded into two branches to synthesize acoustic–phonetic and phonotactic information, optimizing the shared module in CNN-based SLI systems for effective feature extraction. Similarly, deep learning frameworks have also been applied in the field of ASR [14,25,26,27,28], with federated graph learning further enhancing distributed data modeling via global-guided adversarial generation [29].

## 3. Proposed Framework

### 3.1. Definition of Filamentary Convolution

For a TFAF $A=\left(A_{1},A_{2},\cdots ,A_{i},\cdots ,A_{N}\right)$, consisting of *N* short-time spectra, filamentary convolution is an operation applied to each short-time spectrum $A_{i}$. For filamentary convolution with *K* consecutive layers, the output of a particular layer is given by

(1) $$S_{i}^{k}=F\left(A_{i}|\theta_{k}\right)=f_{k}\left(\cdots f_{2}\left(f_{1}\left(A_{i}|\theta_{1}\right)|\theta_{2}\right)|\cdots |\theta_{k}\right),$$

where k∈[1,K], and $\theta_{i}$ represents the parameters of the *i*-th layer. The convolution operation for the *k*-th layer can be represented as

(2) $$S_{i}^{k}=f_{k}\left(S_{i}^{k-1}|\theta_{k}\right)=h\left(W_{k}⊗A_{i}+b_{k}\right),$$

where $W_{k}$ and $b_{k}$ represent the weights and bias in layer *k*.

Thus, the final output feature from multiple filamentary convolution layers is $S=\left(S_{1},S_{2},\cdots ,S_{i},\cdots ,S_{N}\right)$. This operation ensures that the feature maintains a constant number of frames in the time domain. According to the definition, filamentary convolution has a similar kernel shape to 1-D convolution. However, in the field of digital signal processing, to the best of our knowledge, 1-D convolution is mainly applied to audio signals or features, operating along the temporal direction. Although attempts have been made regarding 1-D convolution in the frequency domain, no one has yet defined it or analyzed its characteristics. Therefore, we utilize the term “filamentary” to describe the operation of convolution focusing on the frequency domain within the short-time spectra for subsequent reference and analysis.

### 3.2. Implementation Structure for Filamentary Convolution

**Figure 2:**
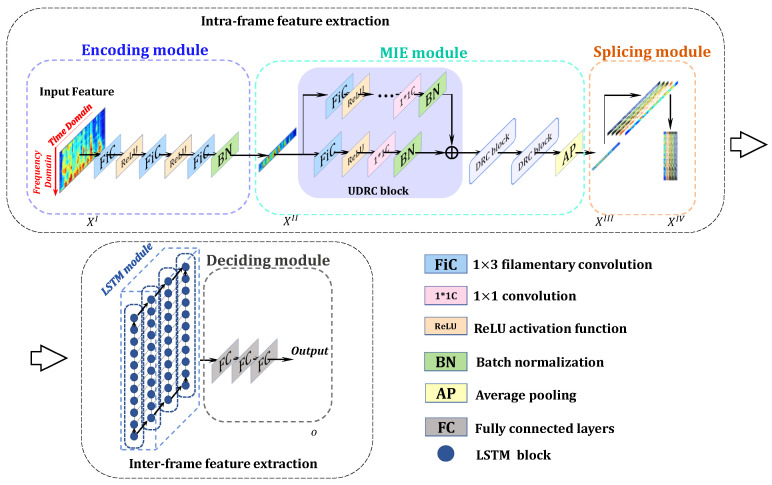
Schematic diagram of the FCK-NN structure.

The CNN-LSTM architecture serves as the baseline system in this work for filamentary convolution implementation and efficiency calculation. Within this framework, we employed the filamentary convolution kernel-based hierarchical neural network (FCK-NN) proposed in our previous work [30], the architecture of which is illustrated in Figure 2, and the details of the model are shown in Table 1.

Our proposed hierarchical language identification system, FCK-NN, is successively composed of an encoding module, multi-level information extraction (MIE) module, splicing module, memory module, and deciding module in the process of forward propagation.

The input audio signal $A\in R^{1\times L}$ is transformed into a TFAF, marked as $X^{I}\in R^{M^{I}\times H^{I}\times W^{I}}$, for the imitation of certain parts of human auditory processing, such as the short-term memory mechanism, parallel processing effect, and multi-level processing mechanism, where $M^{I}$ is the number of channels, and $H^{I}$ and $W^{I}$ are the height and width (the range spanning time and frequency) of each feature map in the TFAF.

We input $X^{I}$ into the encoding module E(·) for a refined output $X^{II}$:

(3) $$X^{II}=E\left(X^{I}\right),$$

where $X^{II}\in R^{M^{II}\times H^{I}\times W^{II}}$ ($W^{II}<W^{I}$) is an encoded feature that ensures that the redundant information is partially removed. The operation in the MIE module M(·) after inputting $X^{II}$ can be described as

(4) $$X^{III}=M\left(X^{II}\right),$$

where the shape of each feature map is fixed in this stage to make the information refined. $X^{III}\in R^{M^{III}\times H^{I}\times W^{III}}$ ($W^{III}<W^{II}$) can be transformed into $X^{IV}\in R^{M^{IV}\times H^{II}}$ in the splicing module S(·) before LSTM:

(5) $$X^{IV}=S\left(X^{III}\right),$$

where $X^{IV}$ is input to the LSTM module L(·) for long-term discrete-time information bridging and the feature extraction of key frames. The output $X^{V}\in R^{c}$ passes through the deciding module:

(6) $$X^{V}=L\left(X^{IV}\right),$$

where c represents the output classes. The result provides scores for languages, where we make decisions regarding the predicted language with the top-1 score.

In conclusion, the input audio is transformed into a TFAF by applying preprocessing. Then, the short-term spectra of the TFAF may be parallelly mapped and transformed in the first three modules, and key frames are successfully extracted via sequential processing by applying LSTM for language identification.

### 3.3. Parallel Processing Within Spectrum

Filamentary convolution operates within the spectra of TFAFs, ensuring parallel operations on each short-time spectrum. This parallel processing maintains feature consistency across different short-term spectra. Due to weight sharing in convolution, each operation behaves akin to a “filter”. More precisely, we employ “filtering-like” to describe this process, because filamentary convolution conducts identical (or parallel) computations on frequency components at different time steps (frames), resulting in one (or multiple) channels of features with consistent input–output sizes. While this resembles the fundamental concept of filtering, “filtering” typically focuses on the removal of specific frequency components, whereas filamentary convolution emphasizes the remapping and encoding of features within the frequency domain. Therefore, it cannot be strictly termed “filtering”.

**Figure 3:**
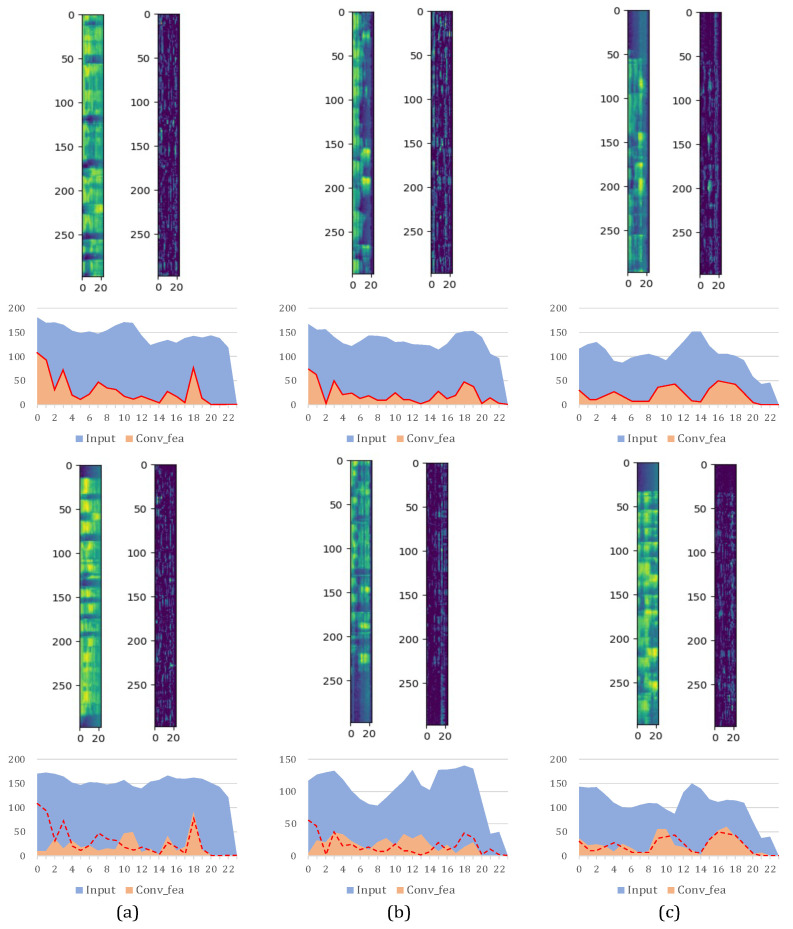
Comparison of features and frequency distributions before and after filamentous convolution over three different languages. Language (**a**) exhibits broad-spectrum energy concentrations (vowel dominance), (**b**) shows fundamental frequency fluctuations (tonal patterns), and (**c**) demonstrates rapid temporal variations (consonant transitions). Intra-language outputs (orange curves per column) cluster distinctively compared to cross-language contrasts.

Given these considerations, the “filtering-like” filamentary convolution offers specialized operations compared to traditional rectangular-shaped convolution or temporal 1-D convolution, particularly emphasizing frequency-domain feature extraction. In Figure 3, we depict the feature maps of three different languages before and after filamentary convolution, alongside their frequency distributions. Horizontally, (a), (b), and (c) represent three distinct languages. Vertically, we compare two different samples in each language. It intuitively reveals that the overall frequency distribution undergoes changes after filamentary convolution. Furthermore, for each language, we compare the frequency distributions after the filamentary convolution of two samples, indicated by red lines corresponding to the upper sample. We observe that the output frequency distributions of samples from the same language tend to be more similar compared to samples from different languages.

The overall frequency distribution visibly alters after filamentary convolution. Moreover, contrasting different languages reveals intuitive variations in their frequency distributions. For instance, the features of language (a) exhibit higher energy values across a broader frequency spectrum, indicating pronounced vowel components and fewer transitions between vowels and consonants compared to the other two languages. Conversely, languages (b) and (c) display more temporal variation in their frequency distributions, signifying greater tonal diversity. Language (b) represents a tonal language; hence, the notable fluctuations in the fundamental frequency (F0) reflect its tonal complexities.

In research on the human auditory system and the human brain, there are also clues suggesting that the phonological and acoustic laws corresponding to visible features in the spectrogram above are part of the language features recognizable by humans. Research [2] indicates that, when humans construct auditory pathways for unfamiliar languages, they initially rely on phonology and intonation to grasp the language’s essence. This indicates why humans can discern a language even when unfamiliar with its vocabulary, solely based on intonation.

**Figure 4:**
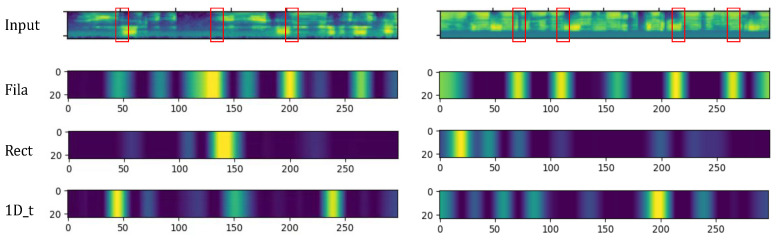
CAM activation maps of input features after filamentary convolution, rectangular-shaped convolution, and 1-D temporal convolution with highlighted critical regions (red boxes).

Following this logic, we attempt to compare different convolutional patterns in extracting relative visible features. We plot CAM activation maps obtained from input features after three different convolutions in Figure 4. The figure visually shows that, after filamentary convolution, there are more activated segments compared to other methods. As highlighted in the red box, it is evident that the features extracted through filamentary convolution exhibit different focal points compared to other methods. This suggests differing underlying mechanisms for low-level speech feature extraction between the three methods. Visually, filamentary convolution captures more information about the frequency variation Within TFAFs. This is attributed to filamentary convolution’s ability to conduct intra-frame parallel feature extraction at the frequency level, thereby preserving inter-frame relationships and facilitating the extraction of inter-frame relationship features in subsequent stages.

### 3.4. Maintaining Details Among Frames

#### 3.4.1. Retaining Detailed Temporal Characteristics

**Figure 5:**
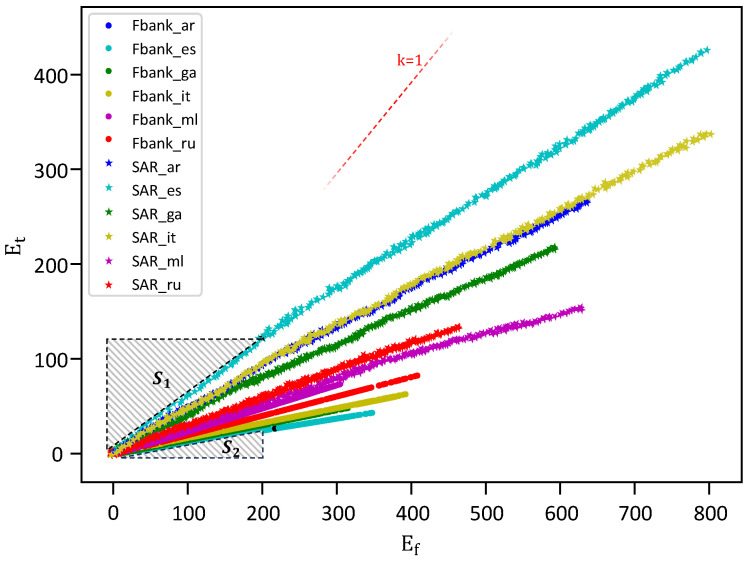
Intra-frame and inter-frame continuity scatter plot for SAR and Fbank.

As mentioned in the previous section, filamentary convolution preserves detailed temporal characteristics by abstaining from inter-frame operations. We contend that inter-frame operations during low-level feature extraction may dilute crucial information. Specifically, it often leads to the premature mixing of key information, thereby diminishing the feature extraction efficiency. As argued in [14], a reduction in temporal resolution is often detrimental. This premature mixing may stem from disparate neighborhood continuities present in TFAFs across temporal and frequency dimensions. Notably, continuities along the frequency axis are strong, and those along the temporal axis are comparatively weaker. Directional information with strong neighborhood continuity tends to have more redundancy; thus, convolution along directions of strong neighborhood continuity may result in higher efficiency. We separately assessed the temporal and spectral neighborhood continuities of both the original speech and TFAF within the graphical representations in Figure 5. Detailed descriptions of the plotting procedures are provided in Section 3.4.2.

Based on the analysis of continuities, it potentially results in reduced computational efficiency when the computations span across frames. Furthermore, empirical evidence, coupled with extensive linguistic research, suggests that linguistic features often do not manifest in adjacent frames but rather emerge at multiple discrete critical moments. This is also why the recognition performance suffers significantly when the duration of speech segments is too short. The non-stationary nature of speech also suggests that the preceding and subsequent moments in a speech signal often lack causal continuity. These pieces of evidence collectively indicate that dogmatically extracting features solely from adjacent frames may not be necessary.

Additionally, from Figure 4, we can intuitively observe that filamentary convolution yields a greater number of high activation values compared to the other two convolution methods. Based on the analysis in the previous section, human language recognition relies more on the variations in frequency features, and filamentary convolution precisely focuses on the changes in frequency features within the speech signal. The characteristics of filamentary convolution allow it to retain more temporal relationships, thereby facilitating the extraction of frequency variation information. For further quantitative verification, we provide statistics of pixels in the output features, focusing on high-level activation values over special thresholds, in Table 2 and Table 3. We selected 20 samples in Chinese and 17,250 test samples covering 44 languages from the test set to plot them, respectively. Among them, we counted the number of output feature values with pixel values greater than 76 (256 × 0.3), 127 (256 × 0.5), and 191 (256 × 0.75). It can be seen that, among the three convolution methods, filamentary convolution yields a higher proportion of high activation values. This indicates its capability to extract more useful information, suggesting that the obtained features provide a better foundation for subsequent temporal feature extraction.

#### 3.4.2. Continuity Evaluation for TFAFs

Raw audio preprocessing yields two-dimensional TFAFs, wherein each dimension manifests distinct continuity levels. To characterize the continuity within each dimension, we introduce $E_{f}$ (intra-frame continuity index in the frequency domain) and $E_{t}$ (inter-frame continuity index in the time domain) for the quantification of neighborhood continuity in this study. Higher values of $E_{f}$ and $E_{t}$ correspond to diminished neighborhood continuity within the source feature.

Let us consider a two-dimensional TFAF denoted as $v\in R^{h\times w}$, where *h* frames and *w* frequency points exist within each frame for feature *v*. The formulations for $E_{f}$ and $E_{t}$ are as follows:

(7) $$E_{f}=\frac{1}{w-1}\frac{1}{h}\sum_{i=0}^{h-1}\sum_{j=0}^{w-2}\sqrt{{\left|v_{ij}-v_{i(j+1)}\right|}^{2}},$$

(8) $$E_{t}=\frac{1}{h-1}\frac{1}{w}\sum_{j=0}^{w-1}\sum_{i=0}^{h-2}\sqrt{{\left|v_{ij}-v_{(i+1)j}\right|}^{2}},$$

where $v_{ij}$ represents the *i*-th frame and *j*-th frequency point in the TFAF *v*.

We introduce the spatial audio representation(SAR) as an alternative to TFAFs to compute $E_{f}$ and $E_{t}$ from raw audio. The SAR constitutes a frame-by-frame representation, where each frame consists of audio clips. To generate an SAR, we employ the same windowing and framing procedures as for TFAFs, specifically utilizing Fbank techniques. We present the continuity analysis between SAR and TFAFs by plotting ($E_{f}$, $E_{t}$) in Figure 5. The plotted samples encompass data from six languages, each comprising 300 audio clips.

The star symbols in the Figure 5 represent Fbank samples, while circular symbols represent SAR samples. All star samples are present on the right side of the reference line with slope k=1, which indicates that the frequency continuity of the Fbank features is higher than the time continuity. SAR exhibits a similar characteristic. Furthermore, we observe that, under equal frequency-domain continuity, $S_{1}>S_{2}$, indicating lesser temporal continuity in SAR, thus rendering this feature sparser overall. The introduction of the STFT enables Fbank to become a relatively suitable feature for subsequent classifier analysis in terms of temporal continuity.

## 4. Experiment

### 4.1. Experimental Setup

#### 4.1.1. Dataset

The selection of appropriate datasets is crucial for language identification tasks, with the quality being assessed based on the data distribution and labeling accuracy [31]. High-quality datasets should maintain a balance between features and ensure consistent mono-labeling for each sample. Balanced feature distribution ensures that the proportion of samples aligns with their occurrence probability in real-world scenarios, while consistent labeling ensures the accurate description of each sample. However, many datasets used in SLI research lack public availability or fail to meet quality standards.

Several datasets have been available in the past or are accessible to specific organizations but not freely available to all researchers. These include the OGI-11L [32] and OGI-22L [33] corpora, the CallHome and CallFriend corpora, the ATR multilingual travel domain speech database, and the Oriental Language Recognition (OLR) [16,34,35,36,37] challenge datasets. Additionally, the NICT 10LAN corpus [38] and the NIST Language Recognition Evaluation corpus [39,40,41], although widely used, are no longer available due to discontinued maintenance.

Researchers without access to open datasets often collect their own data. For example, data may be sourced from websites or recorded in controlled environments. However, datasets collected in this manner may suffer from quality issues such as excessive noise or incorrect labeling. These datasets are typically categorized as either “clean” or “not clean”, with clean datasets recorded in controlled environments and not clean datasets gathered from various sources without quality control.

To address the need for high-quality experimental datasets, we focus on three open corpora: OpenSLR [42], Voxlingua107 [43], and Common Voice 6.1 [44] (details are described in Appendix A.1.1). However, these datasets may not necessarily be conducive to training a better, more generalizable model. OpenSLR, while containing a large amount of audio data, only covers a limited number of languages. Additionally, the data quality may not meet the SLI task requirements. The Voxlingua107 and Common Voice datasets aim to remove invalid data from ample collected audio samples, but their processing strategies may not guarantee clear voice recordings or sufficient durations for SLI tasks.

To address the need for a high-quality dataset for the SLI task, we develop a data refining method called Refining with Double Check (RDC), which employs our proposed examiner–referee refinement strategy. This strategy ensures objective and fair data selection based on specific criteria. We recruited a total of 31 participants, intending to divide them into 20 examiners and 11 referees (10 regular referees and 1 special referee). Thus, each speech sample was assessed by three participants (2 examiners and 1 regular referee). In cases where regular referees were uncertain about a sample, the special referee was consulted for arbitration. We evenly distributed the screening tasks among the over 30 participants to ensure a balanced workload.

The RDC method involves selecting and refining recordings from the OpenSLR and Common Voice datasets. Our refined corpus comprises 100,717 recordings spanning 44 languages, offering a more effective dataset for SLI research.

A recording is deemed valid if it meets three criteria: clear pronunciation, a duration exceeding 3 s, and a muted duration of less than half of the total duration. To ensure fairness, we employ the examiner–referee strategy, where two examiners make initial selections. If they reach a consensus, no further assessment is needed. Otherwise, a referee makes the final decision on validity.

**Figure 6:**
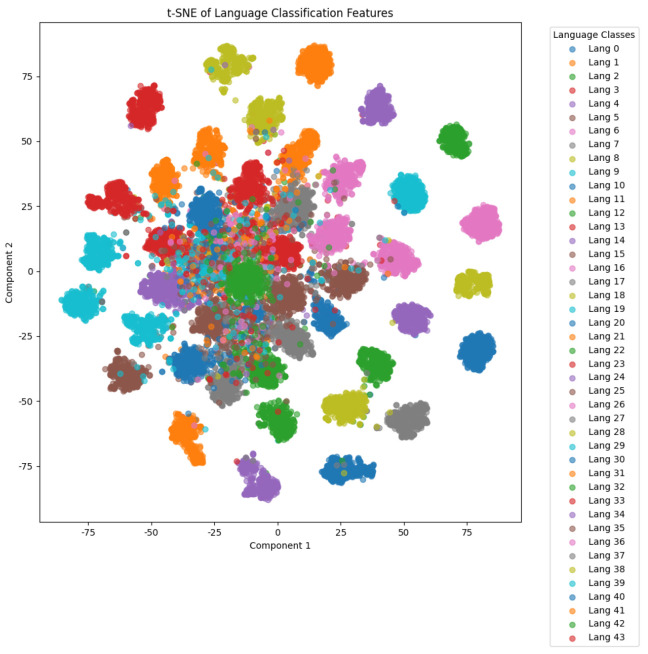
Data distribution for our dataset.

The corpus covers 44 languages, with the names and abbreviations listed in Table 4. Recordings marked with ∗ are from OpenSLR, while the rest are from Common Voice. Training, validation, and testing sets are randomly selected at 60%, 20%, and 20%, respectively, for each language, with all audio files standardized to a 48kHz sample rate. Furthermore, we present the t-SNE distribution for our dataset using FCK-NN in Figure 6.

Filtering improved the recognition performance within the dataset and enhanced its generalization to other datasets. Our strategy retains clear samples while eliminating poor ones, resulting in improved overall performance compared to other datasets. This makes our dataset a more suitable basis for language recognition system performance evaluation.

To further emphasize the advantages of our curated dataset, we conducted cross-validation on multiple datasets shown in Table 5, summarizing each dataset’s characteristics in Table 6. We selected the intersection languages from these datasets as subsets for a fair comparison of these datasets under uniform standards. Upon examining the results in Table 5, we can observe that our dataset exhibited the best performance under self-validation. In comparison to other datasets, our data distribution appeared more concentrated. However, it is important to note that such concentration does not necessarily indicate a high-quality dataset, as it may have the hidden problem of poor generalization. Fortunately, the outstanding performance of our dataset in cross-validation indicates its superior generalizability, thereby facilitating the better learning of linguistic knowledge by neural networks.

In order to explore the reasons behind the improved generalizability, we reviewed our selection strategy and the characteristics of each dataset (as outlined in Table 6). Through this retrospective analysis, we found that our data curation process effectively removed poor and unclear samples, thereby enhancing the validity of the dataset. Specifically, we retained “not clean” data—recordings potentially captured in varying environmental conditions using different devices. These recordings may contain subtle noise from different environments. While such noise does not affect the model’s judgment, it aids in improving the model’s generalization abilities.

#### 4.1.2. Baseline System

We propose a baseline system to validate the effectiveness of the CNN-LSTM framework (Table 7). By integrating our filamentary convolution into the basic CNN-LSTM architecture, we conduct simple ablation studies and validation tests. Furthermore, the separable structure (which separately handles spatial and temporal feature learning) enables efficiency evaluation (parameter utility) across different convolution strategies, supported by a new efficiency metric introduced in the following section.

#### 4.1.3. Incremental Efficiency

We introduce the concept of an *incremental model* to describe a model with more parameters than the base system within the CNN-LSTM framework. For instance, FCK-NN is considered one such incremental model. To assess the efficiency of these incremental models, we propose the concept of *incremental efficiency* (IE) based on the base model $\theta_{0}$. The parameter set for the incremental model $\theta_{k}$ can be represented as $A=\{x_{k}|x_{k}\in \theta_{k},\;x_{k}\;\mathrm{is}\;\mathrm{trainable}\}$, and its number of parameters can be computed by

(9) $$P\left(\theta_{k}\right)=\sum_{x_{i}\in A}count\left(x_{i}\right).$$

It is important to note that we do not employ a pre-train strategy on either the base model or incremental models; thus, $P(\theta_{0})$ and $P(\theta_{k})$ represent the total numbers of parameters in the base model $\theta_{0}$ and the incremental model $\theta_{k}$, respectively.

Therefore, the IE can be defined as

(10) $$IE_{\theta_{0},\theta_{k}}=\lambda \times \frac{Acc^{\theta_{k}}-Acc^{\theta_{0}}}{P\left(\theta_{k}\right)-P\left(\theta_{0}\right)},$$

where $Acc^{\theta_{k}}$ and $Acc^{\theta_{0}}$ denote the accuracy achieved by models $\theta_{k}$ and $\theta_{0}$, respectively. λ is a scale coefficient used to regulate IE within a suitable range.

This index intuitively quantifies our expectations for efficient networks: higher IE values indicate a higher utilization rate of network parameters. Essentially, IE represents the ratio between accuracy and the number of parameters, specifically focusing on the difference in values between the incremental model and the base model.

#### 4.1.4. TFAF Generation

Our model requires an appropriate input representation, with a few representative TFAFs including Fbank, MFCC, and PLP. These representations can be generated from raw audio using open frameworks such as Kaldi (https://kaldi-asr.org/), as in our experiments. Details of generation are outlined in Table 8:

These TFAFs represent information in two dimensions. The range of the time dimension is determined by the duration of the raw audio clip, window length, and frame length. The generated TFAFs are of the same size within the time domain since they are consistent in these factors. However, there are differences in the frequency domain due to the implementation of different operations, leading to different TFAFs. In our case, we use the default settings provided by Kaldi to generate these TFAFs. Additionally, voice activity detection operation is not utilized during generation because we have found that the original pauses and gaps in speech signals play a positive role in language identification.

### 4.2. Ablation Evaluation

#### 4.2.1. Experiments on Different Input Features

As demonstrated in Table 9 and Table 10, Fbank exhibits better compatibility with *incremental models* in our experiments. In our experimental setup (using the default settings in Kaldi to generate MFCC, PLP, Fbank), Fbank tends to contain more frequency information due to the minimal compression of the frequency scale. More finely processed TFAFs help to alleviate the classifier’s burden by filtering out and discarding unimportant information through deliberate design. However, these more refined TFAFs do not necessarily perform well in models that excel in terms of low-level feature extraction capabilities. Therefore, the experimental results demonstrate the phenomenon, as shown in Table 9.

#### 4.2.2. Experiments on Voice Activity Detection

Voice activity detection (VAD) is a preprocessing method for speech signals, with the core objective of distinguishing effective speech segments from non-speech segments (e.g., silence or background noise) in audio signals, thereby optimizing system performance. This method is integrated into the Kaldi framework. After VAD processing, silence portions can be removed, and, during the generation of TFAFs, users can manually configure whether to apply VAD to the speech signals. To identify the optimal SLI strategy, we conducted ablation experiments to validate the impact of VAD on the SLI task. For this purpose, we constructed two subsets: one containing samples with long silence durations and the other derived from our dataset. The former subset was sampled from the raw dataset (noise-free), differing from the latter only in retaining samples where effective speech occupied less than half of the duration. Thus, we labeled the former as low-speech-ratio samples and the latter as high-speech-ratio samples. We ensured that both subsets had identical sample sizes (matched class counts and equal samples per class).

Under this setup, we compared the classification performance of the two subsets with and without VAD, as summarized in Table 11. The results indicate that, for the low-speech-ratio subset, using VAD reduces the classification error rates, which aligns with the conclusions of most prior work. However, for the high-speech-ratio subset, disabling VAD slightly improves the SLI task performance. A plausible explanation is that the silence segments in high-speech-ratio samples primarily stem from linguistic pause patterns, which may inherently serve as discriminative features for language identification.

#### 4.2.3. Experiments on Components of CNN-LSTM Framework with Filamentary Convolution

We conduct ablation experiments on the filamentary convolution-based FCK-NN with Fbank input. The results in Table 12 demonstrate that each module is essential for FCK-NN in establishing a hierarchical feature extraction framework. Additionally, removing the MIE module results in a more severe decline in accuracy than removing the encoding module. This indicates that the MIE module is vital in capturing abundant language features in FCK-NN. Furthermore, we conduct ablation experiments over routes in the UDRC block. As shown in Table 12, we test UDRC with different combinations of three routes (details are illustrated in our previous work in [30]). The final components of UDRC are chosen to be the shallow route and the deep route, where two 1×3 layers are reserved in the deep route. In brief, we select the final FCK-NN structure for language identification tested after our ablation experiments, and most of our visual validations of filamentary convolution in the preceding sections were accomplished using this structure’s FCK-NN.

#### 4.2.4. Experiments on Filamentary Convolution

For the FCK-NN framework, the conclusion is that convolution implemented with filamentary-shaped kernels (filamentary convolution) within the frequency domain performs better than convolution implemented with square-shaped kernels (rectangular-shaped convolution) or 1-D convolution within the time domain (1-D temporal convolution). Specifically, filamentary convolution is more efficient than 1-D temporal convolution, and filamentary convolution aids the model in achieving higher accuracy compared to 1-D temporal convolution. With the established framework shown in Figure 2, we evaluate the performance by alternating convolution with filamentary convolution, 1-D temporal convolution, and rectangular-shaped convolution.

The kernel sizes can be formalized as $1\times k_{1}+1\times k_{2}$, $k_{1}\times 1+k_{2}\times 1$, and $k_{1}\times k_{1}+k_{2}\times k_{2}$. $k_{1}$ represents the convolution size in the encoding module, and $k_{2}$ represents the convolution size in the MIE module. As shown in Table 13, filamentary convolution with 1×2+1×3 achieves the highest accuracy. It is pertinent to note that the performance is related to the overlapping strategy in the encoding module. For the non-overlapping strategy, the stride aligns with the kernel shape in the encoding module, e.g., $1\times k_{1}$ corresponds to $\left(1,k_{1}\right)$ stride. Filamentary convolution achieves better accuracy only if $k_{1}=2$ with the non-overlapping strategy. For this strategy, convolution can be considered as a filter bank over the spectrum. The value 2 represents the period of the filter bank within each operation. The conclusion can be drawn that, with the proper period, the entire system may perform better.

Filamentary-shaped kernels notably improve the efficiency. From an overall perspective, the experimental results do not unequivocally prove that filamentary convolution is always better than rectangular-shaped convolution in terms of accuracy. However, for the IE index, filamentary convolution shows an impressive improvement, as shown in Table 10 and Table 13, indicating that feature extraction using traditional rectangular-shaped convolution cannot fully optimize the utilization of the parameters.

In contrast, the performance of networks with 1-D temporal convolution is inferior to that of those with filamentary convolution or rectangular-shaped convolution in terms of both accuracy and IE (λ is set to 1 in our experiments), as shown in Table 13. Specifically, bridging cross-frame features during low-level feature extraction is obviously incompatible with the FCK-NN framework. Another piece of evidence is that the larger the kernel size that 1-D temporal convolution implements, the greater the decline in accuracy and efficiency with the non-overlapping strategy. Therefore, proper feature extraction within the frequency domain helps LSTM to capture language features precisely and efficiently.

Additionally, the results in Table 10 support the above conclusions, where we verify our findings across 1 s and 3 s audio clips and their three different TFAFs. Furthermore, we have validated the above conclusions by varying the sampling rate. The results indicate that our previous conclusions still hold.

### 4.3. Cross-Method Comparison

#### 4.3.1. Comparison Among SLI-Based Systems

Above all, our FCK-NN is evaluated against previous studies using CNN-based SLI systems, as shown in Table 14. The evaluations are conducted over the large-scale dataset created by ourselves among different TFAFs. In particular, for Fbank, FCK-NN shows the capacity for low-level information extraction.

Moreover, the CRNN-Inception framework, with a structure for the stacking of outputs from layers with differently sized convolution kernels, is adept in capturing multi-level comprehensive information. However, FCK-NN provides more detailed multi-level information extraction through filamentary convolution in UDRC blocks. It may improve the SLI performance with a more compatible design. Moreover, the LID-Senones framework stands out due to its convolution spanning the whole frequency domain for feature extraction. The effectiveness of LID-Senones helps to confirm our finer frequency information capture.

#### 4.3.2. Further Validation Among Other Acoustic-Based Models

As a convolutional framework, filamentary convolution offers a perspective for the analysis of speech signals. Consequently, it should not be confined solely to the CNN-LSTM framework. For further validation, we extensively selected classification models incorporating convolutional operations for further experimentation, as shown in Table 15. One category combined convolution with temporal extraction modules (including ECAPA-TDNN [25] and campplus [26]), while the other consisted solely of convolution (including ResnetSE [27], PANNS_CNN10 [46], ERes2Net [47], and Res2net [48]).

The results from the models in the former group (upper part of the table) indicate that filamentary convolution, when coupled with temporal modules, can yield performance improvements, with higher efficiency compared to square convolution. Specifically, we utilize convolution as a preliminary feature extractor, accumulating it within the corresponding model. The computational efficiency of this accumulation is termed the accumulation efficiency, which can be calculated using Equation (10). $\theta_{0}$ is the original model, and $\theta_{k}$ denotes the model accumulating filamentary convolution or 1-D time convolution. Our choice of accumulation strategy is due to models such as ECAPA-TDNN, which lack a square convolutional structure. Consequently, we propose to accumulate additional convolution at the top end of the original model. The accumulation section acts as a preliminary feature extractor to verify the collaborative performance of convolution and the TDNN temporal extraction module, assessing the impacts of different convolutional patterns on the final performance. We observe that, compared to square convolution, incorporating filamentary convolution leads to higher efficiency in terms of the performance improvement. This also indirectly demonstrates that filamentary convolution, when combined with modules capable of extracting temporal information, enables the superior extraction of primary features.

The validation of the models in the latter group (lower part of the table) suggests a noticeable performance decline with 1-D time convolution. The latter methods contains only convolution modules without temporal extraction modules. The default setting for these convolutions is square convolution. Therefore, we focus on verifying the performance changes brought about by replacing convolution with filamentary convolution and 1-D time convolution. We find that filamentary convolution exhibits less performance degradation. Since the deployment of filamentary convolution and 1-D time convolution results in the same model parameter quantity, the performance degradation per unit parameter induced by temporal one-dimensional convolutions is more pronounced. We represent this degradation coefficient (DC), indirectly confirming the superiority of filamentary convolution. Corresponding to the IE parameters, DC can also be calculated using the formula of IE, namely Equation (10), only substituting $\theta_{k}$ with the original model and $\theta_{0}$ with a model that replaces square convolution with filamentary convolution and 1-D time convolution. Furthermore, we observed that, in frameworks lacking temporal feature extraction modules, filamentary convolution may not necessarily enhance the performance over square convolution because these frameworks lack structures such as LSTM, RNNs, TDNNs, etc., to handle temporal dependencies. The effectiveness of filamentary convolution lies in providing these temporal feature extraction modules with a more efficient frequency representation.

## 5. Conclusions and Future Work

This paper introduces a novel convolutional pattern, “filamentary convolution”, aimed at preserving temporal relationships in time–frequency joint features more effectively. Inspired by the processing mechanism of the human auditory system during speech analysis, filamentary convolution simplifies and enhances the traditional square convolution framework. It achieves this by focusing on parallel frequency-level feature extraction, akin to how humans process speech segments within short-term memory intervals.

In our comparative analysis, filamentary convolution outperformed traditional convolution and 1-D temporal convolution in capturing frequency feature variations in speech. By prioritizing frequency-level features and avoiding the mixing of inter-frame information, filamentary convolution retains more temporal relationship information, thus facilitating subsequent model feature extraction.

Through validation, we confirmed the efficacy of filamentary convolution as a frontend for other systems, leading to highly efficient performance improvements. This approach offers a promising avenue for enhanced speech analysis and related tasks.

Furthermore, filamentary convolution allows for the more direct retrieval of the original positions in the speech sequence that correspond to key information, a capability that is beyond the reach of conventional convolutions. It provides researchers with a fresh perspective on the parallels and distinctions between human perception and neural networks’ processing of critical information.

## Figures and Tables

**Table 1:** Details of the FCK-NN system.

Module	Unit	Layer	Input_Size	Output_Size
Encoding	Convolution	Conv_1	50 × 1 × 298 × 23	50 × 16 × 298 × 12
		Conv_2	50 × 16 × 298 × 12	50 × 64 × 298 × 7
		Conv_3	50 × 64 × 298 × 7	50 × 128 × 298 × 4
		Conv_4	50 × 128 × 298 × 4	50 × 128 × 298 × 4
MIE	UDRC1_Shallow	Conv_1	50 × 128 × 298 × 4	50 × 256 × 298 × 4
		ReLu	50 × 256 × 298 × 4	50 × 256 × 298 × 4
		BatchNormal	50 × 256 × 298 × 4	50 × 256 × 298 × 4
	UDRC1_Deep	Conv_1	50 × 128 × 298 × 4	50 × 256 × 298 × 4
		ReLu	50 × 256 × 298 × 4	50 × 256 × 298 × 4
		Conv_2	50 × 256 × 298 × 4	50 × 256 × 298 × 4
		ReLu	50 × 256 × 298 × 4	50 × 256 × 298 × 4
		Conv_3	50 × 256 × 298 × 4	50 × 256 × 298 × 4
		BatchNormal	50 × 256 × 298 × 4	50 × 256 × 298 × 4
	UDRC2	/	50 × 512 × 298 × 4	50 × 256 × 298 × 4
	UDRC3	/	50 × 256 × 298 × 4	50 × 128 × 298 × 4
	Pooling	Avg_Pool	50 × 128 × 298 × 4	50 × 128 × 74 × 1
LSTM	LSTM	LSTM	50 × 128 × 74	50 × 128 × 50
Decision	Full_Connect	FC_1	50 × 6400	50 × 64
		FC_2	50 × 64	50 × 128
		FC_3	50 × 128	50 × 256
		FC_4	50 × 256	50 × 44

**Table 2:** Number and proportion of pixels with activation values over threshold for 20 Chinese samples, where bold values denote the highest activation counts/proportions under each threshold, emphasizing the superior performance of filamentary convolution.

	Threshold (T) (T/256 %)	Convolution Type		
		Filamentary	1D_t	Rectangular
Amount ($\times 10^{4}$)	76 (30%)	**3.54**	3.27	3.33
	127 (50%)	**2.23**	2.05	2.05
	191 (75%)	**1.22**	1.04	1.13
Proportion (%)	76 (30%)	**25.87**	23.91	24.35
	127 (50%)	**16**	15	15
	191 (75%)	**8.91**	7.61	8.26

**Table 3:** Number and proportion of pixels with activation values over the threshold for 17,250 samples in test set, where bold values denote the highest activation counts/proportions under each threshold, emphasizing the superior performance of filamentary convolution.

	Threshold(T) (T/256 %)	Convolution Type		
		Filamentary	1D_t	Rectangular
Amount ($\times 10^{7}$)	76 (30%)	**3.16**	2.99	2.90
	127 (50%)	**1.93**	1.90	1.75
	191 (75%)	**1.01**	0.99	0.93
Proportion (%)	76 (30%)	**26.7**	25.3	24.6
	127 (50%)	**16.3**	16.0	14.8
	191 (75%)	**8.5**	8.4	7.89

**Table 4:** Details of the corpus proposed, where recordings marked with ∗ are from OpenSLR, while the rest are from Common Voice.

Language	Code	Amount			
		Total	Train	Test	Validation
Arabic	ar	2246	1350	400	400
Breton	br	1979	1200	350	350
Bu-Ninkada *	bu *	2065	1250	400	400
Catalan	ca	2894	1700	550	550
Hakha Chin	cn(cnh)	2831	1700	550	550
Chuvash	cv	2948	1750	550	550
Welsh	cy	2138	1300	400	400
German	de	2331	1400	450	450
Dhivehi	dv	2282	1350	450	450
English	en	2346	1400	450	450
Esperanto	eo	2312	1750	550	550
Spanish	es	2320	1400	450	450
Estonian	et	2309	1400	450	450
Basque	eu	2314	1400	450	450
Persian	fa	2309	1400	450	450
French	fr	2233	1300	400	400
Irish	ga	2814	1700	550	550
Gujarat *	gu *	2334	1400	450	450
Interlingua	ia	2352	1400	450	450
Iban *	ib *	1779	1050	350	350
Indonesian	id	2256	1350	450	450
Italian	it	2257	1350	450	450
Japanese	ja	2323	1400	450	450
Javanese *	jv *	1701	1050	300	300
Khmer *	km *	2287	1350	450	450
Kannada *	kn *	1760	1050	350	350
Slovenian *	si *	2033	1200	400	400
Latvian	lv	2317	1400	450	450
Malayalam *	ml *	2316	1400	450	450
Mongolian	mn	2112	1300	400	400
Nepali *	ne *	1540	900	300	300
Dutch	nl	2277	1350	450	450
Portuguese	pt	2278	1350	450	450
Russian	ru	2338	1400	450	450
Sakha	sa(sah)	2977	1750	550	550
Sovenian	sl	2275	1350	450	450
Sundanese *	su *	1563	950	300	300
Swedish	sv	2970	1750	550	550
Tamil	ta	2326	1400	450	450
Telugu *	te *	2324	1400	450	450
Turkish	tr	2297	1350	450	450
Tatar	tt	2330	1400	450	450
Uighur *	yg *	2332	1400	450	450
Chinese	zh	2392	1450	450	450

**Table 5:** Results of cross-validation on different training and testing subsets using simple CNN-LSTM framework.

Training Subset	Testing Subset				Avg Acc
	Ours	Raw	SLR100	Voxlingua	
Ours	**0.913**	0.908	0.785	0.803	**0.852**
Raw	**0.893**	0.875	0.753	0.806	0.832
SLR100	0.865	0.853	**0.882**	0.782	0.845
Voxlingua	0.735	0.723	0.766	**0.859**	0.771

**Table 6:** Summary of characteristics over various datasets. “Clear” refers to speech segments with distinct pronunciation and intelligibility (even in the presence of ambient noise, as long as linguistic features remain discernible), whereas “Clean” specifically denotes pure speech recorded in controlled environments without background noise. The key distinction lies in the fact that the former focuses on speech intelligibility (intelligibility), while the latter emphasizes the environmental purity (purity) of recordings.

Dataset	Consistent Mono-Label	Clear	Clean	Public Availability
Common Voice	✓			✓
Voxlingua	✓			✓
OpenSLR	✓	✓	✓	✓
NIST LRE	✓	\	\	\
Ours	✓	✓		✓

**Table 7:** Details of baseline CNN-LSTM system.

Unit	Input_Size	Output_Size
Convolution_1	50 × 1 × 298 × 23	50 × 16 × 100 × 8
ReLu	50 × 16 × 100 × 8	50 × 16 × 100 × 8
Convolution_2	50 × 16 × 100 × 8	50 × 64 × 34 × 3
ReLu	50 × 64 × 34 × 3	50 × 64 × 34 × 3
Convolution_3	50 × 64 × 34 × 3	50 × 128 × 12 × 1
BatchNormal	50 × 128 × 12 × 1	50 × 128 × 12 × 1
LSTM	50 × 128 × 12	50 × 128 × 50
Full_Connect_1	50 × 6400	50 × 64
Full_Connect_2	50 × 64	50 × 128
Full_Connect_3	50 × 128	50 × 256
Full_Connect_4	50 × 256	50 × 44

**Table 8:** Generation method and data type of each feature.

Feature	Size	Data Type	Source
MFCC	(298,13)	Float32	Kaldi
PLP	(298,12)	Float32	Kaldi
Fbank	(298,23)	Float32	Kaldi

**Table 9:** Comparison of TFAFs with different convolution strategies.

Feature	Kernel Size		
	1 × 2 + 1 × 3	2 × 1 + 3 × 1	2 × 2 + 3 × 3
MFCC	76.18	64.85	81.58
PLP	87.75	80.80	89.30
**Fbank**	**92.38**	**83.53**	**91.18**

**Table 10:** Efficiency evaluation for FCK-NN with different TFAFs.

Method	Kernel Size	Database			Period	Params (M)	Acc (%)	Base		IE ↑
		Fbank	PLP	MFCC				Params (M)	Acc (%)	
Frequency	1 * 2 + 1 * 3	✓			1 s	2.191	79.89	0.577	60.03	**12.309**
					3 s	2.444	92.38	0.337	76.57	**7.501**
			✓		1 s	1.462	76.90	0.577	69.96	**7.851**
					3 s	1.502	87.75	0.579	82.41	**5.791**
				✓	1 s	1.782	61.40	0.577	50.87	**8.742**
					3 s	1.822	76.18	0.579	67.15	**7.262**
Time	2 * 1 + 3 * 1	✓			1s	3.305	69.20	0.577	60.03	3.365
					3 s	3.306	83.53	0.337	76.57	2.344
			✓		1 s	2.895	61.73	0.577	69.96	–3.550
					3 s	2.896	80.80	0.579	82.41	–0.694
				✓	1 s	2.486	43.81	0.577	50.87	–3.703
					3 s	2.487	64.85	0.579	67.15	–1.207
Both	2 * 2 + 3 * 3	✓			1s	3.389	80.40	0.577	60.03	7.248
					3 s	3.390	91.18	0.337	76.57	4.786
			✓		1 s	3.801	75.76	0.577	69.96	1.800
					3 s	3.806	89.30	0.579	82.41	2.136
				✓	1s	3.596	61.33	0.577	50.87	3.465
					3 s	3.601	81.58	0.579	67.15	4.775

**Table 11:** Comparison of error rates across VAD and VAD-free strategies.

Dataset	Low-Speech-Ratio Samples		High-Speech-Ratio Samples	
	VAD	VAD-Free	VAD	VAD-Free
FCK-NN	0.101	0.110	0.080	**0.076**
LID_Senones [12]	0.168	0.173	0.158	**0.156**

**Table 12:** Ablation evaluation for FCK-NN.

	CNN	LSTM	UDRC			ACC(%) ↑
			a	b	c	
1		✓	✓	✓	✓	87.11
2	✓		✓	✓	✓	80.68
3	✓	✓	✓			88.31
4	✓	✓		✓		89.06
5	✓	✓			✓	75.03
6	✓	✓	✓	✓	✓	90.31
7	✓	✓				72.19
8	✓	✓	✓		✓	88.15
9	✓	✓		✓	✓	89.11
10	✓	✓	✓	✓		**92.38**

**Table 13:** Evaluation of different convolution strategies.

Kernel Shape	Method	Kernel Size	Overlap	Params (M)	Acc (%)	IE ↑
Square	Both	2 × 2 + 3 × 3	Y	4.427	89.71	3.213
			N	3.390	91.18	4.785
		3 × 3 + 3 × 3	Y	4.268	90.59	3.566
			N	3.437	85.00	2.718
		4 × 4 + 3 × 3	Y	4.333	90.91	3.588
			N	3.500	85.00	2.664
Filamentary	Time	2 × 1 + 3 × 1	Y	3.319	80.31	1.255
			N	3.306	83.53	2.344
		3 × 1 + 3 × 1	Y	3.328	80.02	1.154
			N	3.314	82.94	2.139
		4 × 1 + 3 × 1	Y	3.337	80.39	1.274
			N	3.323	80.99	1.480
	Frequency	1 × 2 + 1 × 3	Y	2.596	88.80	**5.413**
			N	2.444	92.38	**7.501**
		1 × 3 + 1 × 3	Y	2.400	88.71	**5.883**
			N	1.626	81.54	**3.858**
		1 × 4 + 1 × 3	Y	2.410	88.85	**5.923**
			N	1.635	88.04	**8.832**

**Table 14:** Experiments on CRNN and LID_Senones against FCK-NN.

Method	Fbank	PLP	MFCC
CRNN [45]	77.58	82.34	69.64
Inception-v3_CRNN [45]	82.49	84.45	72.38
LID_Senones [12]	84.56	79.68	73.24
**Ours**	**92.38**	**87.75**	**76.18**

**Table 15:** Validation of filamentary convolution across other different frameworks.

Method	Original	Param (M)	Fila	Params (M)	IE ↑	Square	Params (M)	IE ↑
ECAPA-TDNN [25]	96.72	6.195	96.85	6.220	**4.813**	96.80	6.271	0.998
campplus [26]	96.87	7.199	97.25	7.224	**14.897**	97.34	7.274	6.219
**Method**	**Original**	**Param (M)**	**Fila**	**Params (M)**	**DC ↓**	**Time**	**Params (M)**	**DC**↓
ResnetSE [47]	96.11	7.821	93.70	5.935	**1.281**	93.58	5.935	1.343
PANNS_CNN10 [46]	95.36	5.244	90.76	2.122	**1.474**	85.73	2.122	3.085
ERes2Net [27]	94.19	6.619	92.59	5.676	**1.690**	90.96	5.676	3.423
Res2net [48]	92.44	5.096	91.54	4.624	**1.909**	89.64	4.624	5.925

**Figure A1:**
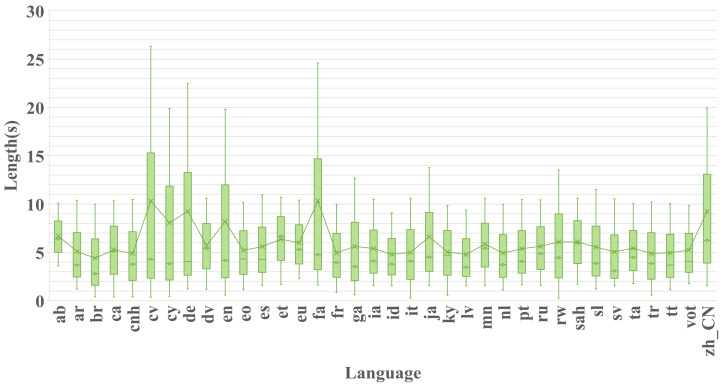
Box figure of each language.

**Figure A2:**
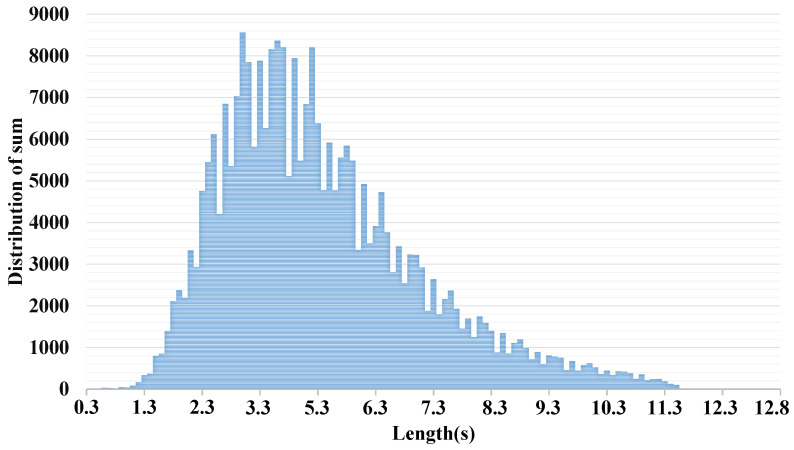
Distribution of durations in our corpus.

**Figure A3:**
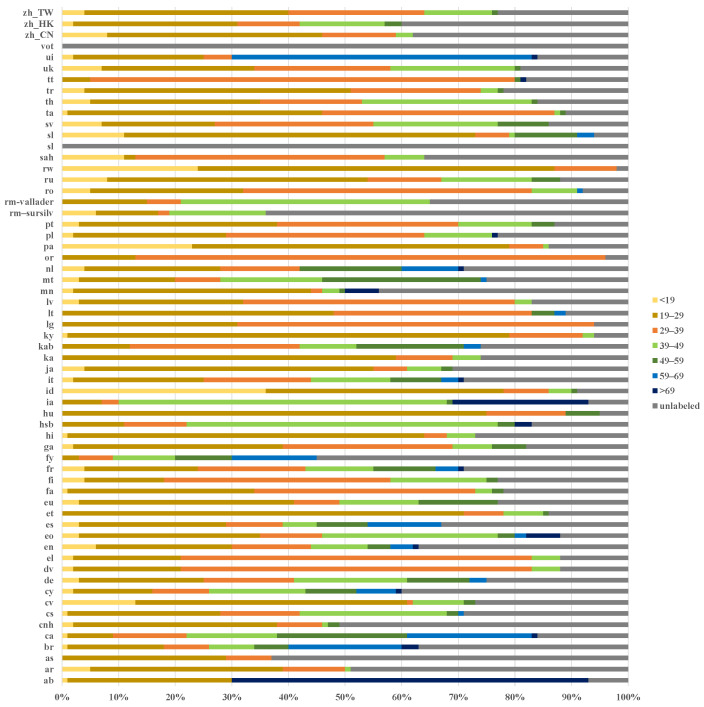
Distribution of age in Common Voice.

**Figure A4:**
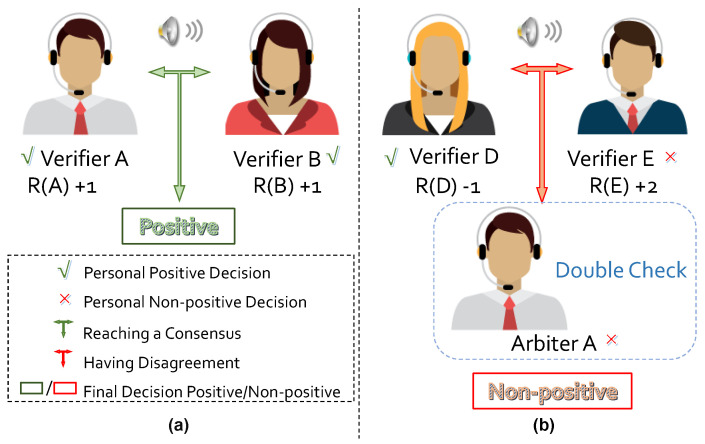
Illustration of refinement with double check strategy, where subfigure (**a**) demonstrates consensus validation (Verifiers A and B with mutual positive evaluations, R(A) +1 and R(B) +1, leading to green "Positive" outcome), while subfigure (**b**) shows disagreement resolution via arbitration (Verifiers D [R(D) -1] vs. E [R(E) +2], resolved by Arbiter A with red “Non-positive” decision), using color-coded interactions (green checkmarks for agreement, red cross for conflict).

**Figure A5:**
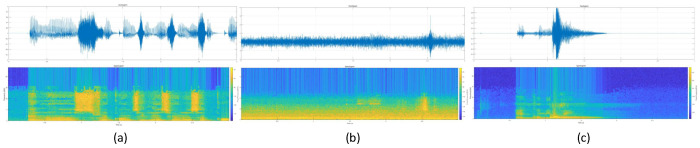
Audio and its spectrogram comparison among (**a**) clean but not clear, (**b**) not clean, and (**c**) clean.

## Data Availability

The source and acquisition method of the data are included in the file, and the specific data content will be made public after subsequent maintenance.

## Appendix A

### Appendix A.1. Raw Data of SLR

#### Appendix A.1.1. Common Voice and OpenSLR

The Common Voice (https://commonvoice.mozilla.org/ (accessed on 7 May 2025)) corpus is a multilingual dataset with transcribed voice clips. English was the only language available when the project was launched in 2017, and other languages started to be used in 2018. Common Voice is completely open and decentralized, which means that anyone can upload their own voice or download its content. Thanks to this project, the lack of sufficient data is no longer an obstacle in NLP (especially in ASR and SLR) engineering and research. The latest version, Common Voice Corpus 6.1, collects voices with 7335 validated hours from 9283 recorded hours in a total of 60 languages. More statistics are elaborated as follows.

First, in Table A1, statistics are provided in terms of the abbreviation (Code), the number of speakers that it contains (Voice), and the total speech hours (Total) and validated speech hours (validation) of each language.

**Table A1:** Data in Common Voice.

Language	Code	Voice	Hours	
			Total	Validation
Abkhaz	ab	14	1	<1
Arabic	ar	672	77	49
Assamese	as	17	<1	<1
Breton	br	157	16	7
Catalan	ca	5376	748	623
Hakha Chin	cnh	297	5	2
Czech	cs	353	45	36
Chuvash	cv	92	16	4
Welsh	cy	1382	124	95
German	de	12659	836	77
Dhivehi	dv	167	19	18
Greek	el	118	13	6
English	en	66173	2181	1686
Esperanto	eo	574	102	90
Spanish	es	19484	579	324
Estonian	et	543	27	19
Basque	eu	1028	131	89
Persian	fa	3655	321	282
Finnish	fi	27	1	1
French	fr	12953	682	623
Frisian	fy	467	46	14
Irish	ga	101	5	3
Hindi	hi	31	<1	<1
Sorbian	hsb	19	2	2
Hungrian	hu	47	8	8
Interlingua	ia	36	8	6
Indonesian	id	219	17	9
Italian	it	5729	199	158
Japanese	ja	235	5	3
Georgian	ka	44	3	3
Kabyle	kab	1309	622	525
Luganda	lg	76	8	3
Lithuanian	lt	30	4	2
Latvian	lv	99	7	6
Mongolian	mn	376	17	11
Maltese	mt	171	15	7
Dutch	nl	1012	63	59
Odia	or	34	7	<1
Punjabi	pa	26	2	<1
Polish	pl	2647	129	108
Portuguese	pt	1120	63	50
Romansh Sursilvan	rm-sursilv	78	9	5
Romansh Vallader	rm-vallader	39	3	2
Romanian	ro	130	9	6
Russian	ru	1412	130	111
Kinyarwanda	rw	410	1510	1183
Sakha	sah	42	6	4
Sovenian	sl	82	7	5
Swedish	sv	222	15	12
Tamil	ta	266	24	14
Thai	th	182	12	8
Turkish	tr	678	22	20
Tatar	tt	185	28	26
Ukrainian	uk	459	43	30
Vietnamese	vi	62	1	<1
Votic	vot	3	<1	<1
Chinese(China)	zh_CN	3501	78	56
Chinese(Hong Kong)	zh_HK	2536	100	50
Chinese(Taiwan)	zh_TW	1444	78	55

Second, we select those languages with a time length of more than 20 h and generate statistics about the duration of each speech clip. The maximum and minimum durations of the speech clips are 36.305 s and 0.264 s, respectively. Figure A2 shows the distribution of the speech clips by time length. We also show the time length of each language in Figure A1. Each box unit contains the upper extreme value, upper quartile value, median value, lower quartile value, and lower extreme value of the audio duration. The points in the line graph show the average values calculated for all audio files in the corresponding language.

Third, a good voice corpus should sample a large number of male and female voice contributors within different age groups. The Common Voice corpus samples 150,757 voice contributors of both genders. As Figure A3 describes, the age of these voice contributors ranges from 0 to 79.

OpenSLR (http://openslr.org/ (accessed on 7 May 2025)), containing 111 independent datasets, includes more than 30 languages. It initially focused on the automatic speech recognition (ASR) field. Thus, most of the datasets contain both speech audio and its transcribed text. The reason that we use this corpus is that it contains some languages that Common Voice lacks. This means that Common Voice has no corresponding language at all or has too few audio files in the corresponding language to train an SLR model. In other words, OpenSLR plays a complementary role.

#### Appendix A.1.2. Dataset Involved in This Experiment

We conduct the experiments with a manually cleaned, multilingual dataset that has more languages than most other datasets. The languages used in this dataset have two sources, namely Common Voice and OpenSLR.

Their audio samples are uneven in duration. However, the input features of the FCK-NN system must be of the same size in the time domain. Therefore, it is imperative to guarantee that all processed speech from the two sources falls within the same duration. Since the target duration is 3 s in our experiment, audio samples shorter than 3 s are abandoned and those longer than 3 s are cut to 3 s clips. The latter strategy abandons audio clips of the same duration at both ends of raw audio but preserves the largest numbers of three audio clips connected in sequence.

The audio samples are also uneven in quality. Some audio samples contained in the two corpora cannot meet the demands of experiments. Analyses of and solutions to these problems are elaborated in the following paragraphs.

Although the audio sets from the Common Voice database have been validated, some noisy audio samples still remain. The uneven audio quality of Common Voice renders it necessary to organize the qualified audio. In the screening process, we disregarded the undesirable audio as follows.

We removed audio with environmental noise, including hoots from cars, the sound of rain, and noise at construction sites; audio with overwhelming white noise, electric sizzle, and high-latency echo; and audio mixed with other voices.

We gathered a new subset of multilingual audio from Common Voice. Each language of this subset contained 3000 audio clips, which were the raw data to be used in the future screening. This means that those languages with less than 3000 audio clips were not used in our subset. After manual inspection, the error rate for 32 languages was 5.3% (160/3000) at most and 1.8% (54/3000) on average. To prove the necessity of audio cleaning, simple experiments were conducted to compare the raw data with the cleaned data in terms of data availability. Using the same data before and after clearing to perform classification across all 32 languages, we found that the accuracy of FCK-NN was improved by over 15%. This proves that the cleaning of Common Voice was necessary and effective.

Our investigation revealed that OpenSLR’s audio files in the selected languages all met the requirements of the prospective corpus. Specifically, these audio files, which were recorded in a quiet environment, had a uniform volume and sample rate. Thus, the audio files available in OpenSLR could be directly used in our corpus only after the cutting process.

#### Appendix A.1.3. Our Proposed Corpus Refining Method

An effective corpus for the SLI task should contain enough valid recordings, which is beneficial for the training of an effective language classifier. To select valid recordings, we set up a criterion to determine whether a given recording was valid or not. In this work, the valid recordings had to contain a clear voice and their duration had to be long enough.

More specifically, we defined valid recordings as those satisfying three requirements. Firstly, the pronunciation of the voice in the recording had to be clear enough to be recognized by the listener. Secondly, the duration had to be more than 3 s. Thirdly, the mute duration had to be less than half of the whole duration.

According to the above data selection criteria, we proposed the *examiner–referee* refinement strategy to guarantee that the data selection is objective and fair. More specifically, we invited two examiners to participate in the determination and data selection. If they reached an agreement on the selection of one valid/invalid recording, an additional referee was not invited to make a final decision. In other cases, the referee determined whether the recording was valid or not. We designed such an *examiner–referee* strategy to reduce the number of participants and guarantee the effectiveness and fairness of the data selection.

We invited *N* persons, one as the referee and the other N−1 as the examiners, to participate in our proposed *examiner–referee* audio data refinement. First of all, one referee had to be selected from the participants, and the referee had to be someone who could make a decision regarding the determination of valid data, ensuring that they were accurate and reliable. To select such a referee, we firstly chose some audio recordings, denoted as G, from the corpus and invited another *L* people to select the valid data from G based on our given selection criteria via voting. The voting result was used to evaluate the accuracy of the data selection of each participant. Then, the recordings of G were assigned to these *N* participants, respectively, and each person selected the valid data from G according to our given criteria. Finally, the person who achieved the highest accuracy was appointed as the referee. The procedure of the *examiner–referee* refinement strategy is summarized in Algorithm A1.

**Algorithm A1** Procedure of corpus refinement.
**1. Generation of determination reference:** (1) Composing the dataset G by randomly selecting *M* audio recordings from all the recordings to be refined; (2) Distinguishing the valid and invalid data of G by the voting of *L* people; (3) Recording the voting result R as a determination reference. **2. Referee selection:** (1) Inviting *N* participants to distinguish the valid and invalid data of G; (2) Comparing the determination result of each participant with R; (3) Selecting the referee who achieves the highest accuracy. **3. Data examination:** (1) Selecting any two different people from the remaining N−1 participants to compose $C_{N-1}^{2}$ examination groups, where the members of each group are denoted as $e_{1}$ and $e_{2}$; (2) Equally dividing the total *K* recordings of corpus C into $C_{N-1}^{2}$ subsets $c_{i}\left(i=1,2,\cdots ,C_{N-1}^{2}\right)$, where each $c_{i}$ consists of $n=K/C_{N-1}^{2}$ recordings; (3) Inviting each group of examiners to examine the validity of the recordings of the corresponding $c_{i}$. For each $c_{i}$: **for** j=1:n **do** Examining $c_{i}\left(j\right)$ by $e_{1}$ and $e_{2}$; **if** $e_{1}$ and $e_{2}$ achieve agreement **then** **if** $c_{i}\left(j\right)$ is invalid **then** Removing $c_{i}\left(j\right)$ from $c_{i}$; **end if** **else** Inviting the referee for final determination; **if** $c_{i}\left(j\right)$ is invalid **then** Removing $c_{i}\left(j\right)$ from $c_{i}$; **end if** **end if** **end for** **4. Corpus refinement:** Refining C with the updated $c_{i}$.

#### Appendix A.1.4. Our Proposed Corpus Refining Method

In this work, we aimed to compose a new corpus that covered over 44 languages and contained enough valid audio recordings for each language. We constructed our dataset based on the Common Voice corpus and the OpenSLR corpus by using our proposed *examiner–referee* refinement strategy. The strategy of double checking is illustrated in Figure A4. In the Common Voice corpus, there exist 60 subsets composed of 60 corresponding languages. Meanwhile, the OpenSLR corpus contains 132 subsets for 33 corresponding languages. According to the characteristics of the Common Voice corpus, we firstly selected $N_{1}$ subsets, where each subset contained over 3000 long-duration recordings for one language, to compose a corpus $C_{1}$. Then, we selected *K* subsets containing over 2000 long-duration recordings and corresponding to another $N_{2}$ different languages from the OpenSLR corpus and added them to $C_{1}$ to compose an updated corpus, denoted as $C_{2}$. Note that $C_{2}$ covers $N_{1}+N_{2}$ languages, and there are at least 2000 recordings for each language. After this, we applied our proposed *examiner–referee* refinement strategy to $C_{2}$ to obtain the corpus $C_{3}$. Finally, we cut each recording of $C_{3}$ into a 3 s clip and used all resulting recordings to construct our corpus.

#### Appendix A.1.5. Characteristics of Clear Samples

To demonstrate the significance of our data screening, we present some of the data in Figure A5. The spectrograms of completely pure audio, clear but not clean audio, and completely noisy audio in the raw dataset are visualized. We can see that the characteristics of the frequency information in the spectrogram of the noisy audio are almost unidentifiable, but the clear but not clean audio can be identified by the human eye. This also shows that the samples of “clear but not clean” data that we screened were valid. Furthermore, we also verified that the sound quality of most of the samples obtained conformed to both situations (a) and (c). Furthermore, regarding example (c), we deleted it during the screening process because, although the quality of the audio met the requirement, the duration of its effective audio was too short and it did not conform to our requirement of “a muted duration of less than half of the total duration”.
